# Prognostic Implications of Deep Muscle Invasion and High Grade for Bladder Urothelial Carcinoma

**DOI:** 10.7759/cureus.10802

**Published:** 2020-10-05

**Authors:** Atif A Hashmi, Sana Rafique, Rimsha Haider, Shahzeb Munawar, Muhammad Irfan, Javaria Ali

**Affiliations:** 1 Pathology, Liaquat National Hospital and Medical College, Karachi, PAK; 2 Internal Medicine, Liaquat National Hospital and Medical College, Karachi, PAK; 3 Emergency Medicine, National Institute of Blood Diseases and Bone Marrow Transplantation, Karachi, PAK; 4 Internal Medicine, Liaquat College of Medicine and Dentistry, Karachi, PAK; 5 Public Health, Baylor Scott & White, Waco, USA; 6 Statistics, Liaquat National Hospital and Medical College, Karachi, PAK

**Keywords:** urothelial carcinoma, bladder cancer, high grade urothelial carcinoma, low grade urothelial carcinoma, deep muscle invasion

## Abstract

Introduction

Urothelial carcinoma (UC) is the most common bladder cancer. The most censorious pathological aspect of UC is deep muscle invasion and tumor grade. In this study, we assessed the prognostic implications of tumor grade and deep muscle invasion in UC.

Methods

It was a retrospective cross-sectional study conducted at the Department of Histopathology, Liaquat National Hospital, from July to December 2019. The data were collected over five years from January 2014 till December 2018. Records from archives of the anatomic pathology were searched, clinical characteristics were recorded, and histopathological slides were reviewed. Histological parameters, including tumor grade and muscle invasion, were evaluated. Records of patient follow-up were assessed by reviewing clinical records. Recurrence of UC and overall survival was also recorded. Multivariate binary logistic regression was applied for variables that were significant on univariate logistic regression. Survival analysis was performed using the Kaplan-Meier method.

Results

The mean age of the patients was 63.39 ± 14.1 years. More than half (53%) cases were of low-grade papillary UC. Disease recurrence was observed in 53 (39.1%) patients, whereas the mortality rate was 16.6%. In our study, 49 (32.5%) patients were found to have deep muscle invasion. By multivariate analysis, we found that the deep muscle invasion was significantly associated with male gender and grade. In addition, a significant association of high-tumor grade with survival status of the patients was noted.

Conclusions

A high proportion of UC cases in our study were found to have deep muscle invasion and high-tumor grade. Moreover, a significant association of deep muscle invasion with tumor grade and an association of tumor grade with survival signify the prognostic value of these factors in UC.

## Introduction

Bladder cancer is among the top 10 cancers of the human body [1]. Urothelial carcinoma (UC) is the most common type of bladder cancer. The most censorious pathological aspect of UC is the presence or absence of invasion, which can be diagnostically challenging at times. Based on the detection of this invasion, UC is divided into invasive and noninvasive UC. This invasion can be limited to lamina propria and muscularis mucosa or it can even go beyond the mucosa to involve muscularis propria (deep muscle). Invasion of muscularis propria, also referred to as detrusor muscle, is clinically most important as this dictates future management [2]. After the invasion, the second most important pathological feature of UC is grade. World Health Organization (WHO) classification of tumors of the urogenital tract divides UC based on histological parameters into low grade and high grade [3]. Beyond invasion and grade, various other biomarkers have been tested in UC [4-6], some of which such as p53, CK20, and androgen receptor [7,8] have shown some promising results. However, to date, their routine use is not recommended by the College of American Pathologists (CAP) or any other international pathological organization.

The purpose of this stratification of UC into various categories based on invasion vs. no invasion and low vs. high grade is to split tumors into prognostic groups so that response to various treatments can be assessed in these different groups. Validation of prognostic significance of these defined groups is necessary in different populations, as tumor biology differs in divergent populations. Therefore, in this study, we evaluated the prognostic significance of these different groups of UC based on grade and muscle invasion in our population.

## Materials and methods

**Figure 1:**
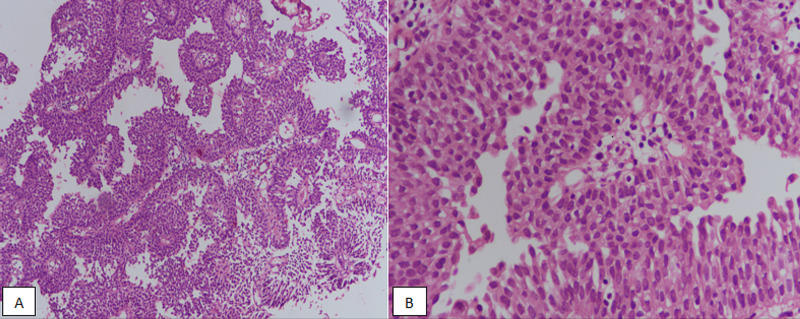
Low-grade urothelial carcinoma. (A) H&E stained sections at 100x magnification showing fused and branching papillae. (B) 400x magnification revealing moderate atypia of tumor cells. H&E, hematoxylin and eosin

**Figure 2:**
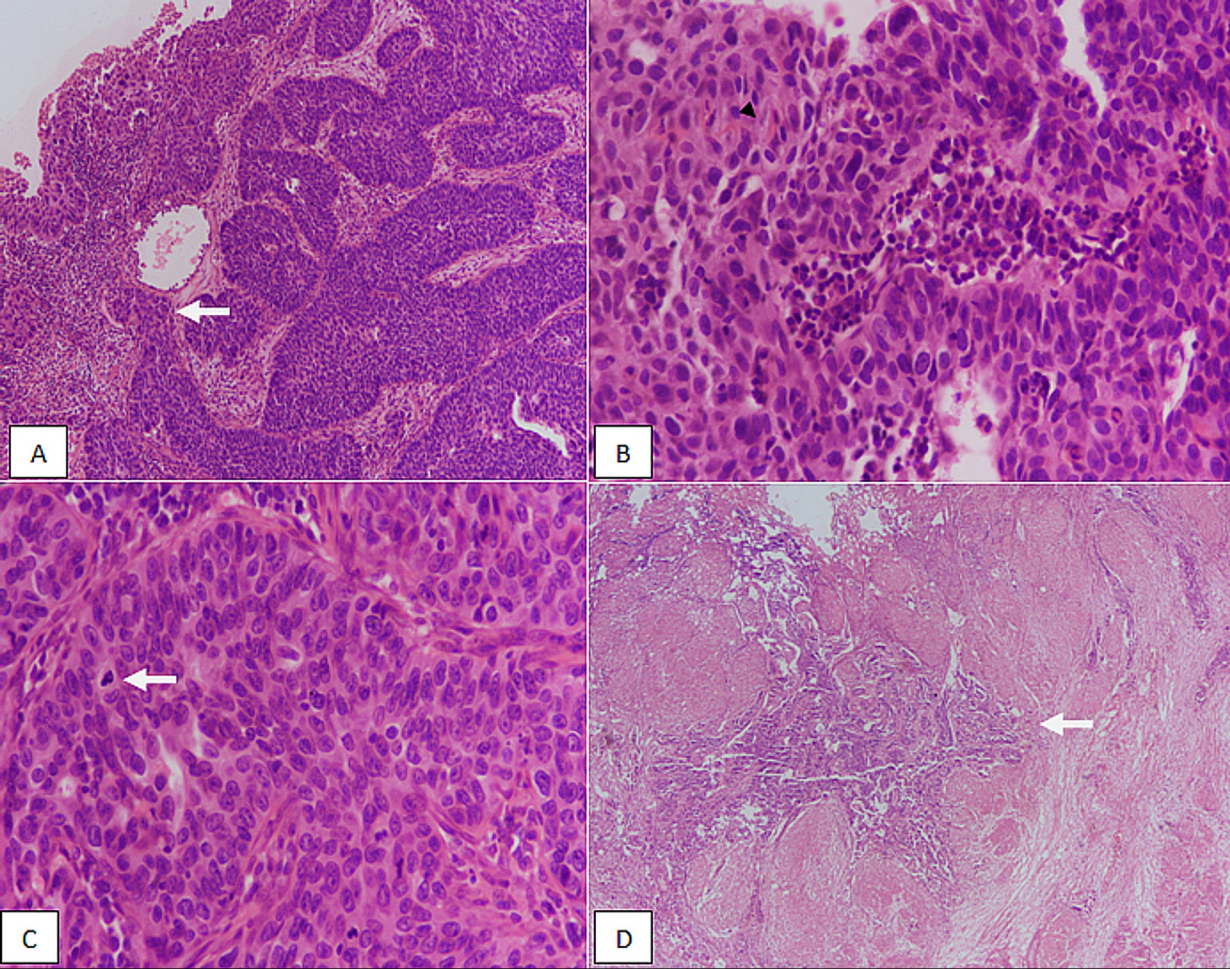
High-grade urothelial carcinoma. (A) H&E sections at 100x magnification showing fused branching papillary architecture. Lamina propria invasion is also noted (arrow). (B) 200x magnification showing loss of polarity. (C) 400x magnification revealing severe atypia and mitosis (arrow). (D) 100x magnification showing deep muscle invasion (arrow). H&E, hematoxylin and eosin

This was a retrospective cross-sectional study conducted at the Department of Histopathology, Liaquat National Hospital, from July to December 2019. The data were collected over five years from January 2014 till December 2018. Records from archives of pathology were searched, clinical characteristics were recorded, and histopathological slides were reviewed. All specimens were received in the histopathology laboratory at Liaquat National Hospital. After the gross examination, representative sections were submitted according to defined criteria. Cases with a pathological diagnosis of UC were included in the study. Cases with the diagnosis of urothelial papilloma or papillary urothelial neoplasm of low malignant potential were excluded from the study. Additionally, patients with any prior history of radiation or chemotherapy were also excluded from the study. Histological parameters, including tumor grade and muscle invasion, were evaluated according to the CAP criteria (Figures 1, 2).

Records of patient follow-up were assessed reviewing clinical records. Recurrence of UC and survival were also recorded.

Statistical analysis

Data analysis was performed using Statistical Package for Social Sciences (SPSS, Version 26.0, IBM Corp., Armonk, NY, USA). Chi-square test was used to check the association. The odds ratio was calculated using univariate and multivariate logistic regression. Multivariate binary logistic regression was applied for variables that were significant on univariate logistic regression. Survival analysis was performed using the Kaplan-Meier method. P-values ≤ 0.05 were considered as significant.

## Results

Clinicopathological characteristics of the population under study

Data of a total of 151 patients were evaluated for this study. The mean age of the patients was 63.39 ± 14.1 years, ranging from 32 years to 92 years. Out of 151 patients, 110 (72.8%) were males and 41 (27.2%) were females. More than half (53%) cases were of low-grade papillary UC. Recurrence was observed for 53 (39.1%) patients, whereas the mortality rate was 16.6%. In our study, 49 (32.5%) patients were found to have deep muscle invasion as presented in Table 1.

**Table 1:** Descriptive statistics of population under study

Characteristics	Frequency (%)
Age (years)	
Mean ± SD	63.39 ± 14.05
≤50 years	38 (25.2)
>50 years	113 (74.8)
Follow-up (months)	24.50 ± 14.53
Gender	
Male	110 (72.8)
Female	41 (27.2)
Specimen type	
Transurethral resection	145 (96)
Radical cystectomy	6 (4)
Tumor grade	
Low grade	80 (53)
High grade	71 (47)
Recurrence	
Yes	59 (39.1)
No	92 (60.9)
Survival status	
Alive	126 (83.4)
Expired	25 (16.6)
Deep muscle invasion	
Present	49 (32.5)
Absent	102 (67.5)

Association of deep muscle invasion with prognostic parameters

We found a significant association of deep muscle invasion with gender (p = 0.014) and tumor grade (p = 0.006), as shown in Table 2.

**Table 2:** Association of deep muscle invasion with clinicopathological parameters Chi-square test was applied *p-Value significant as < 0.05

Clinicopathological parameter		Deep muscle invasion, frequency (%)		p-Value
		Present (n = 49)	Absent (n = 102)	
Gender	Male	42 (85.7)	68 (66.7)	0.014*
	Female	7 (14.3)	34 (33.3)	
Age group	≤50 years	14 (28.6)	24 (23.5)	0.504
	>50 years	35 (71.4)	78 (76.5)	
Tumor grade	Low grade	18 (36.7)	62 (60.8)	0.006*
	High grade	31 (63.3)	40 (39.2)	
Recurrence	Yes	23 (46.9)	36 (35.3)	0.170
	No	26 (53.1)	66 (64.7)	
Survival status	Alive	40 (81.6)	86 (84.3)	0.678
	Expired	9 (18.4)	16 (15.7)	

By univariate analysis, we found that male patients were more likely to have the deep muscle invasion in comparison to female patients. Patients with low-grade papillary UC were found less likely to have the deep muscle invasion compared to patients with high-grade papillary UC; patients who have recurrence of disease were found more likely to have the deep muscle invasion compared to patients with no disease recurrence. By multivariate analysis, we found that gender and grade were significantly associated with deep muscle invasion, as shown in Table 3.

**Table 3:** Odds ratios by univariate and multivariate binary logistic regression for muscle-invasive urothelial carcinoma Univariate and multivariate binary regression were applied *p-Value significant as < 0.05 **Reference group CI, confidence interval

Clinicopathological parameter		Univariate analysis		Multivariate analysis	
		Odds ratio (95% CI)	p-Value	Odds ratio (95% CI)	p-Value
Gender	Male	3.0 (1.22-7.37)	0.017*	3.02 (1.20-7.57)	0.018*
	Female**	1		1	
Age group	≤50 years	1.30 (0.60-2.80)	0.504	NA	-
	>50 years**	1			
Tumor type	Low grade	0.37 (0.18-0.75)	0.006*	0.37 (0.18-0.76)	0.007*
	High grade**	1		1	
Recurrence	Yes	1.62 (0.81-3.24)	0.171	NA	-
	No**	1			
Survival status	Alive	0.82 (0.33-2.03)	0.678	NA	-
	Expired**	1			

**Figure 3:**
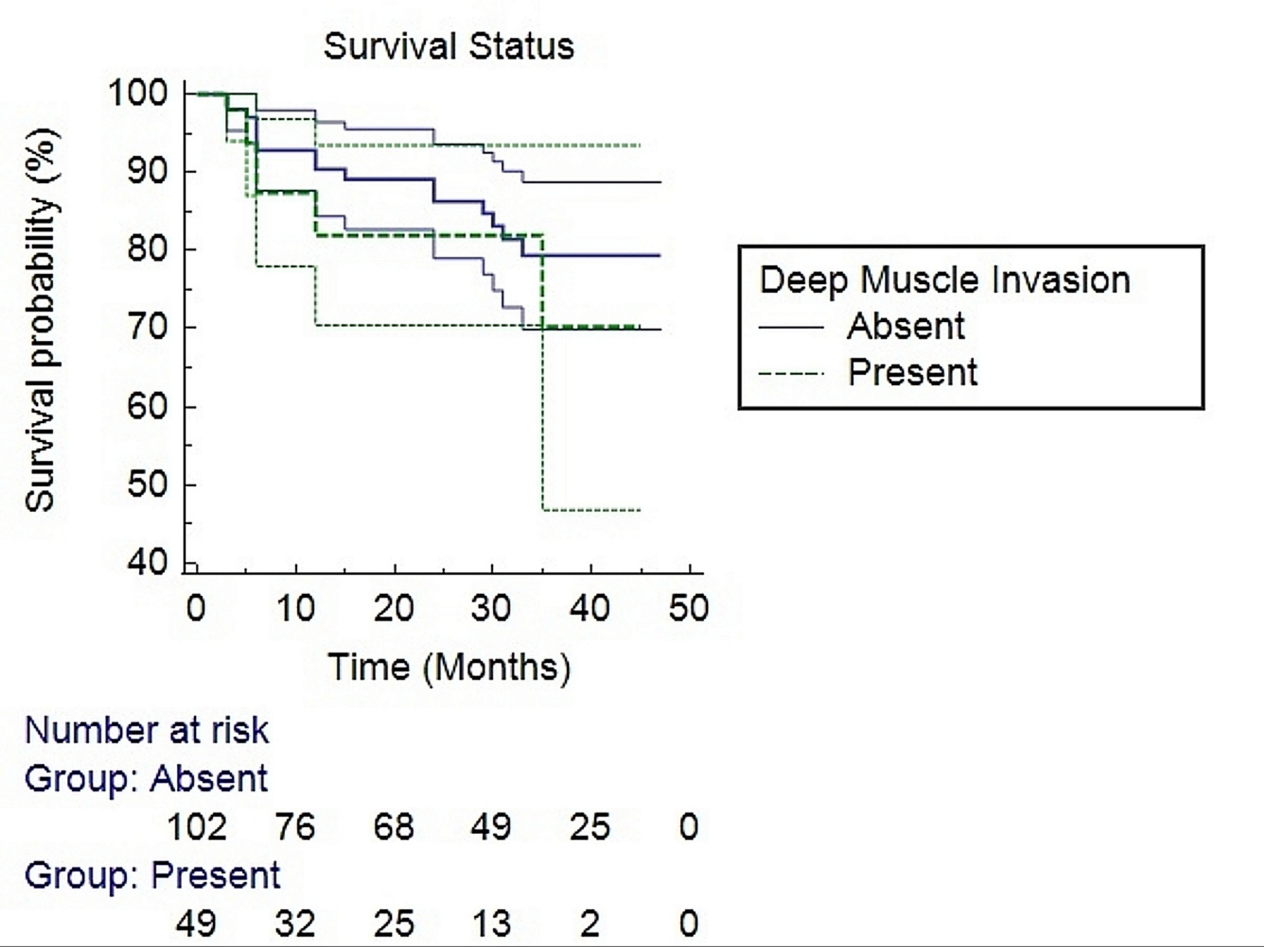
Survival analysis using the Kaplan-Meier method for deep muscle invasion

We assessed survival of the patients with bladder carcinoma and plotted Kaplan-Meier curves to evaluate the association of deep muscle invasion with overall survival. No significant association of deep muscle invasion with overall survival was seen (p = 0.442) in our study (Figure 3).

Association of tumor grade with prognostic parameters

A significant association of tumor grade was noted with lamina propria invasion, deep muscle invasion, and overall survival (Table 4).

**Table 4:** Association of tumor grade with clinicopathological parameters Chi-square test was applied *p-Value significant as < 0.05

Clinicopathological characteristics		Tumor grade Frequency (%)		p-Value
		Low grade (n = 80)	High grade (n = 71)	
Gender	Male	57 (71.3)	53 (74.6)	0.639
	Female	23 (28.8)	18 (25.4)	
Age group	≤50 years	30 (37.5)	29 (40.8)	0.674
	>50 years	50 (62.5)	42 (59.2)	
Lamina propria invasion	Present	4 (5)	36 (50.7)	<0.0001*
	Absent	76 (95)	35 (49.3)	
Recurrence	Yes	30 (37.5)	29 (40.8)	0.674
	No	50 (62.5)	42(59.2)	
Deep muscle invasion	Present	18 (22.5)	31 (43.7)	0.006*
	Absent	62 (77.5)	40 (56.3)	
Survival status	Alive	73 (91.3)	53 (74.6)	0.006*
	Expired	7 (8.8)	18 (25.4)	

By univariate analysis, patients with lamina propria invasion and deep muscle invasion were more likely to have high tumor grade than those without lamina propria and deep muscle invasion. Similarly, patients who had disease recurrence were also more likely to have high-grade UC. By multivariate analysis, a significant association of tumor grade was noted only with survival status of the patients (Table 5).

**Table 5:** Odds ratios by univariate and multivariate binary logistic regression for high-grade urothelial carcinoma Univariate and multivariate binary regression were applied *p-Value significant as < 0.05 **Reference group CI, confidence interval

Clinicopathological characteristics		Univariate	p-Value	Multivariate	p-Value
		Odds ratio (95% CI)		Odds ratio (95% CI)	
Gender	Male	1.188 (0.578-2.444)	0.640	NA	
	Female**	1			
Age group	≤50 years	0.422 (0.194-0.919)	0.030*	0.375 (0.139-1.015)	0.053
	>50 years**	1		1	
Lamina propria invasion	Present	19.54 (6.454-59.174)	0.0001*	21.93 (6.52-73.76)	-
	Absent**	1		1	
Recurrence	Yes	1.15 (0.598-2.215)	0.674	NA	-
	No**	1			
Survival status	Alive	0.28 (0.11-0.72)	0.009*	0.23 (0.084-0.68)	0.008*
	Expired**	1		1	
Deep muscle invasion	Present	0.37 (0.185-0.757)	0.006*	0.81 (0.31-2.06)	0.659
	Absent	1		1	

**Figure 4:**
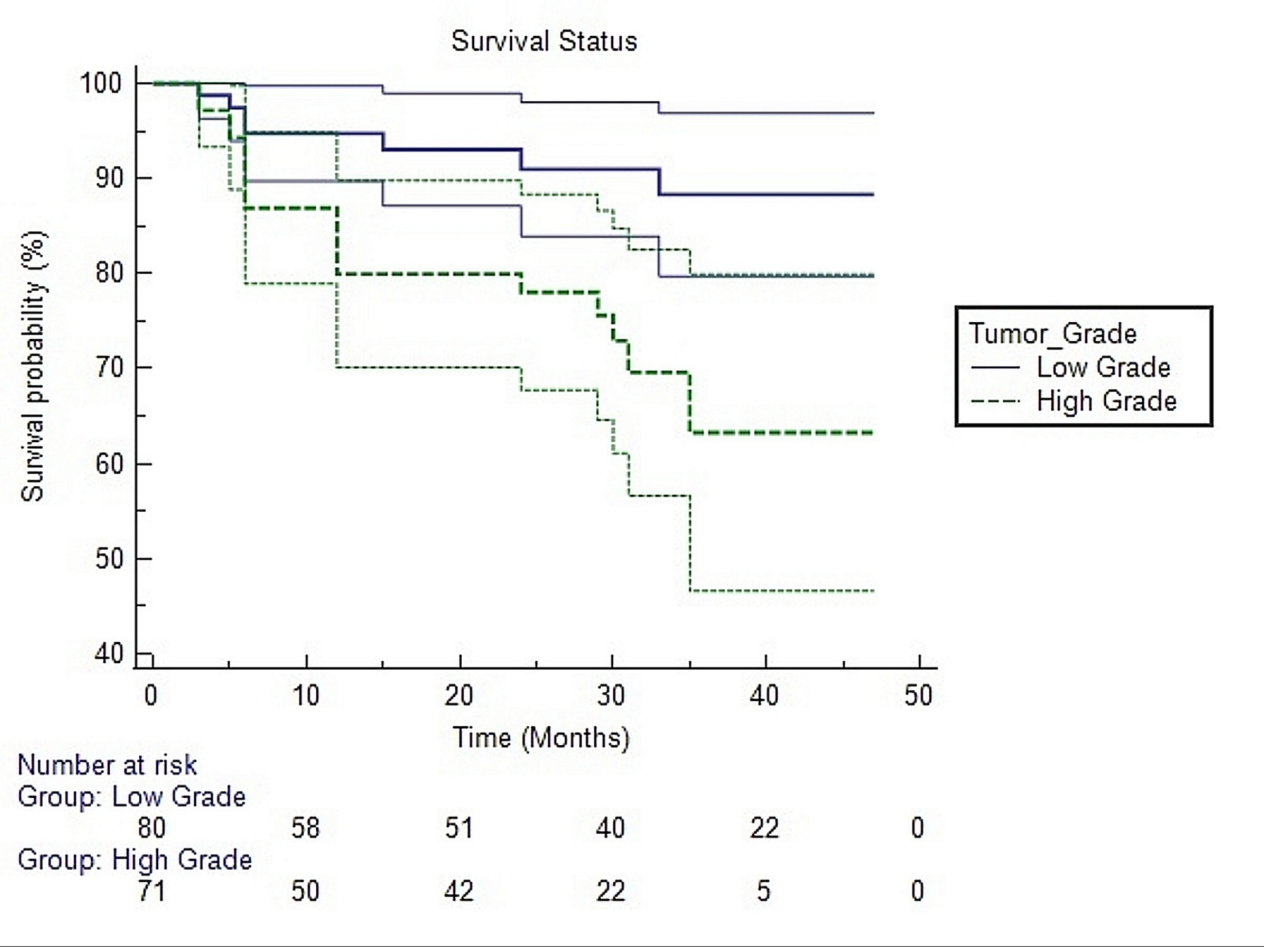
Survival analysis using the Kaplan-Meier method for tumor grade

We evaluated the association of tumor grade with overall survival by plotting Kaplan-Meier curves and found a significant association of tumor grade with overall survival (p = 0.005), as shown in Figure 4.

## Discussion

In this study, we assessed the association of tumor grade and deep muscle invasion with various prognostic parameters by univariate and multivariate analysis and found a significant association of deep muscle invasion with gender and grade. Alternatively, high tumor grade was found to be significantly associated with survival status by multivariate analysis.

Several studies have evaluated prognostic parameters in bladder cancers. A meta-analysis involving 33 studies and 19,702 patients concluded that high tumor grade and deep muscle invasion (tumor stage) were associated with cancer-specific survival [9]. Concordant with these findings, we also noted a significant association of tumor grade with overall survival. Conversely, by multivariate analysis, we did not find any significant association of deep muscle invasion with overall survival. This lack of association in our study can be due to the limited number of cases. An interesting finding in our study was the association of male gender with deep muscle invasion. Although we did not evaluate the impact of male gender on survival for bladder cancer in our study, these sex differences in cancer-specific survival were also reported in a large German multi-center study involving about 2,500 patients with bladder cancer [10].

Some studies have also reported the prognostic significance of lymphatic and vascular invasion in bladder cancer. Muppa et al. also delineated the predictive value of lymphatic and vascular invasion for nodal metastasis in bladder cancer [11]. In our study, we did not assessed lymphatic and vascular invasion as most of the specimens were of transuretheral resection rather than radical cystectomies.

Early diagnosis of any cancer is key to improved prognosis. In developed countries like the United States, the prognosis of prostate cancer has improved after the implementation of prostate specific antigen (PSA) testing based screening programs. Moreover, if PSA levels are high, the threshold for prostate biopsy is low, and that leads to early detection of prostate cancer, resulting in better prognosis. Unfortunately, in underdeveloped countries, like Pakistan, there are no such screening protocols, and due to this patients present late, leading to poor overall prognosis. Hence, there is an immense need to implement screening protocols in our country to improve the prognosis of patients with prostate cancer.

We acknowledge a few limitations to our study, as retrospective studies are inherently subjected to some biases. The sample size of our study was small, and the number of cases of radical cystectomies was limited.

## Conclusions

In this study by multivariate analysis, we a found significant association of deep muscle invasion with male gender and high grade. Alternatively, high-tumor grade was significantly associated with survival status.
